# Market landscape and insurer–provider integration: the case of ambulatory surgery centers

**DOI:** 10.1093/haschl/qxae081

**Published:** 2024-06-11

**Authors:** Xiaoxi Zhao, Michael R Richards, Cheryl L Damberg, Christopher M Whaley

**Affiliations:** RAND Corporation, Santa Monica, CA 90401, United States; Jeb E. Brooks School of Public Policy, Cornell University, Ithaca, NY 14853, United States; RAND Corporation, Santa Monica, CA 90401, United States; RAND Corporation, Santa Monica, CA 90401, United States; Department of Health Services, Policy & Practice, School of Public Health, Brown University, Providence, RI 02903, United States

**Keywords:** vertical integration, health care provider, insurer, ambulatory surgery center

## Abstract

Insurer–provider integration is a new form of vertical integration, with increasing prominence in health care markets. While there are potential benefits from tighter alignment between providers and payers, risks of perverse impacts on health care markets loom large. Yet, little is known about this new wave of consolidation, which limits options for policy or regulatory responses. We focus on a dominant insurer's acquisitions of ambulatory surgery centers (ASCs) to document the growth and geographic spread of these ownership events. We found that a diverse swathe of the United States has experienced an insurer-led ASC takeover. The acquisitions are also more frequently in areas where the insurer holds a higher enrollee market share at baseline, although a linear prediction of the likelihood of ASC acquisition shows a more nuanced picture.

## Introduction

Health care market consolidation in the United States has evolved over time. Historically, consolidation consisted primarily of horizontal mergers between hospitals and, to a lesser extent, physician practices.^1,2^ These types of consolidation have both attracted scrutiny from regulators and met limitations around the number of remaining independent providers to acquire. Starting around 2010, consolidation trends shifted towards hospital–physician vertical integration. Over the last decade, the share of physicians either directly employed by a hospital or health system, or employed by a practice organization acquired by a hospital or health system, has approximately doubled.^3,4^ In addition to private equity investments, the most recent iteration of provider ownership is being driven by insurers directly acquiring providers.^5^

The full extent of insurer–provider integration and its impacts are not well understood. Particularly in capitated payment models, this form of vertical integration has the potential to fully link clinical and financial incentives to provide appropriate and high-quality care, which could benefit patients. High-performing health systems such as Kaiser and Geisinger have long vertically integrated providers and insurers under the same organization.^6,7^ While insurer–provider integration raises concerns that acquired providers may limit care or raise prices for rival insurers, little data exist on this form of consolidation, its extent and scope, as well as its impacts. These knowledge gaps limit the ability of regulators and policymakers to determine whether policy action is warranted.

To begin shedding new light on this topic, we examined insurer acquisition of 1 type of provider, specifically ambulatory surgery centers (ASCs). Approximately 5500 ASCs operate nationwide and are a common setting for outpatient surgical and diagnostic procedures.^8^ Ambulatory Surgery Centers are a growing target for insurer acquisition as public and private payers increase incentives to move care from more expensive settings (ie, hospitals) to less expensive settings of care. We particularly focus on acquisition of ASCs by UnitedHealthcare (UHC) and its subsidiary Optum, given that UHC is the leading insurer acquiring provider practices and has the largest number of employed or affiliated physicians of any entity in the country, totaling 90 000 physicians.^9,10^

**Figure 1.**
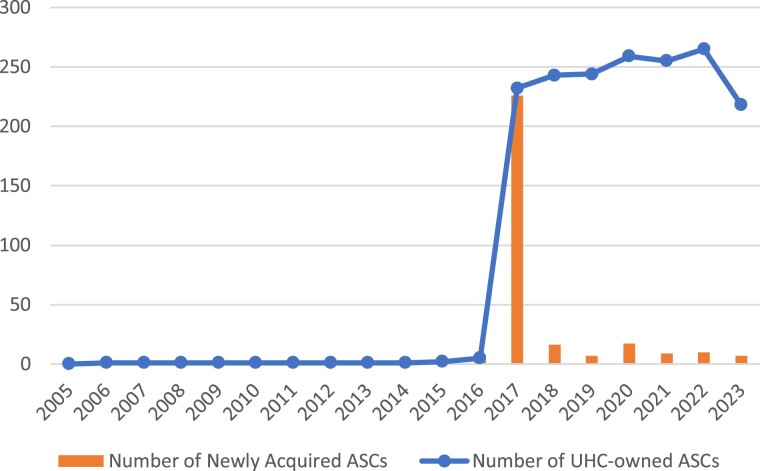
Annual trends in UnitedHealth acquisition of ambulatory surgery centers. Source: Authors' analysis of data on ASC ownership obtained from an FOIA request and UnitedHealth SEC 10-K fillings on relevant business subsidiaries. Abbreviations: ASC, ambulatory surgery center; FOIA, Freedom of Information Act; SEC, Securities and Exchange Commission; UHC, United HealthCare.

As shown in Figure 1, growth in the number of UHC-owned ASCs was driven by Optum's 2017 acquisition of Surgical Care Affiliates (SCA)—one of the largest ASC chains spanning over 30 states.^11,12^

While UHC is a national brand, it is not necessarily the case that its acquisitions have been evenly spread across the United States. We examined the geographic spread of these market events from 2005 to 2020 and, relatedly, explored if certain health care market characteristics correspond to United/Optum ASC ownership. Doing so can help illuminate potential business strategies underlying this newest phase of US health care merger and acquisition (M&A) dealmaking and raise awareness for certain markets that state and/or federal regulators may need to monitor going forward.

Researchers, industry stakeholders, and policymakers have sounded alarms regarding the substantial and increasing M&A activity in the US health care system and its potential consequences. The Biden administration has likewise spotlighted consolidation in markets as a key policy concern and has signaled a desire to temper greater consolidation across numerous sectors, including health care. However, without more supporting data, a lot of key questions and considerations will remain unanswered, which makes it difficult to craft an optimal policy response.

## Data and methods

### The Securities and Exchange Commission filings on subsidiaries

We sought to identify acquisition of ASCs by UHC and describe factors associated with acquisitions. UnitedHealthcare is a large company with numerous subsidiaries, and the direct ownership may not be under the name of UHC or its primary subsidiary, Optum. To identify acquisitions by UHC and its numerous subsidiaries, we need information on other UHC subsidiaries. We used UHC's 10-K Securities and Exchange Commission (SEC) filings from 2014 to 2023^13^ to obtain the names of UHC subsidiaries that substantially contribute to UHC's overall business. According to those 10-K reports the total number of UHC subsidiaries increased from 344 to 2193 in all business sectors from 2014 to 2023.

### The Trilliant Health data on health care providers

We used the Trilliant Health provider data to restrict our UHC subsidiaries to health care providers owned by UHC and to obtain their National Provider Identifiers (NPIs).^14^ The Trilliant Health provider data are based on their all-payer national claims data and validated with the National Plan and Provider Enumeration System (NPPES) data and the Physician Compare data. The claims data have been used in other peer-reviewed articles.^15,16^ Using fuzzy name matching, we identified 1239 providers including ASCs and other types of providers such as home health agencies that were owned by UHC for at least 1 year between 2014 and 2023.

### Data on ASC ownership

We supplemented these data with data on ownership of ASCs obtained through a Freedom of Information Act (FOIA) request to the Centers for Medicare and Medicaid Services.^17^ We first attributed ASC owners with “UnitedHealthcare” and “Optum” in their names as UHC owners. We also combined the SEC-Trilliant data with FOIA data through NPIs to obtain ASCs' Medicare enrollment IDs. As the FOIA and SEC–Trilliant data contain ownership information in different years, our study sample represents the union of the 2 data sources. We implemented a similar approach for SCA-owned ASCs, using SCA's 2016 10-K filing. After the matching, we identified 296 ASCs that were owned (partially) by UHC for at least 1 year from 2005 to 2020, including 209 ASCs that were previously owned by SCA.

### Data on insurer markets

We used data from the Decisions Resource Group (now owned by Clarivate) on county-level insurance enrollment by insurance carrier. The data provided the enrollment counts across payers and insurance types and were generated based on national surveys and company-specific web resources.^18^ We identified UHC's market share for both commercial and public payer (eg, Medicare Advantage and Medicaid managed care) populations.

### Data on ASC markets

We utilized a 100% sample of 2013–2020 Medicare fee-for-service (FFS) claims data to construct measures of outpatient surgery market concentration. We used counties to define geographic markets and separately measured market concentration by type of providers—ASC and hospital outpatient department (HOPD)—using the Herfindahl-Hirschman Index (HHI). Herfindahl-Hirschman Indexes are used by researchers and regulators to measure market competition and range from close to 0 in competitive markets to 10 000 in markets dominated by a single firm. Individual ASC or HOPD market shares, which contribute to the HHI calculations, were calculated using the annual number of procedures performed in the Medicare FFS data.^19^

### Supplementary data

In the ASC-level analyses, we further used the Medicare FFS claims data to construct ASC-level measures of the annual number of procedures performed, annual number of Medicare beneficiaries treated, and patient mix. The measures are linked with the data on the UHC acquisitions via facility Medicare enrollment IDs and tax IDs. We additionally incorporated broader county-level characteristics, such as population, metropolitan status, and poverty level.

### Methods

We first examined descriptive patterns of UHC acquisition of ASCs. We focused on both the national trend in ASC ownership over time and the geographic spread. Next, we documented the correspondence between UHC's local insurance market presence and the health care provider market where the ASC acquisitions occurred. From a strategic standpoint, acquiring ASCs could facilitate further market dominance for UHC within markets, or alternatively, allow UHC to be better positioned for future growth in markets where they currently have limited insurance presence.

We focused our analysis on all UHC ASC acquisitions and compared the UHC's county-level insurance market share in counties with and without an acquired ASC in the year prior to the acquisition. We complemented this analysis with a parallel approach for ASC and HOPD market structures. Specifically, we investigated if acquired ASCs operated in a market with higher HHI concentration for ASCs or HOPDs to speak to potential provider-side strategic gains from UHC ownership.

We completed our analysis with an ASC-level multivariate analysis to further examine the correlation between UHC ASC acquisitions and the characteristics of insurer and provider markets. More specifically, we restricted our sample to the year right before the acquisition for UHC ASCs and all non-UHC ASCs. In addition to the UHC insurance market share and measures of provider market concentration, we also included individual ASC characteristics and market-level demographics in the regression.

### Limitations

This study has limitations. First, we focused on acquisition of a single provider type, ASCs. While ASCs are an emerging target of insurer acquisition and a setting for many outpatient surgeries, other evidence highlights growing prevalence of insurer acquisition of physician practices and direct employment of physicians that also warrants examination. This study also focuses on a single, albeit the nation's largest, insurer. Future work should explore acquisitions by other less market-dominant insurers. Our analysis used patient-level Medicare FFS data to examine patterns of ASC acquisition; it is possible that acquisition decisions are made in response to patient differences among Medicare Advantage and commercial populations that are not captured in these data.

Finally, the aim of this study is to provide policy-relevant information on the patterns of insurer–provider acquisition for a common provider of outpatient surgeries. Future research is needed to examine the factors in acquisition decision and the impacts of this form of integration on provider prices, patient access to care, and care quality.

## Results

### Descriptive trends in ASC acquisition

As previously noted, Figure 1 presents the aggregate trend in UHC-owned ASCs over the 2005 to 2023 period. These acquisitions include both direct acquisitions by UHC, as well as acquisitions by UHC subsidiaries. The purchase of SCA by Optum in 2017 corresponds to the sharp increase in acquired ASCs in Figure 1.

**Figure 2.**
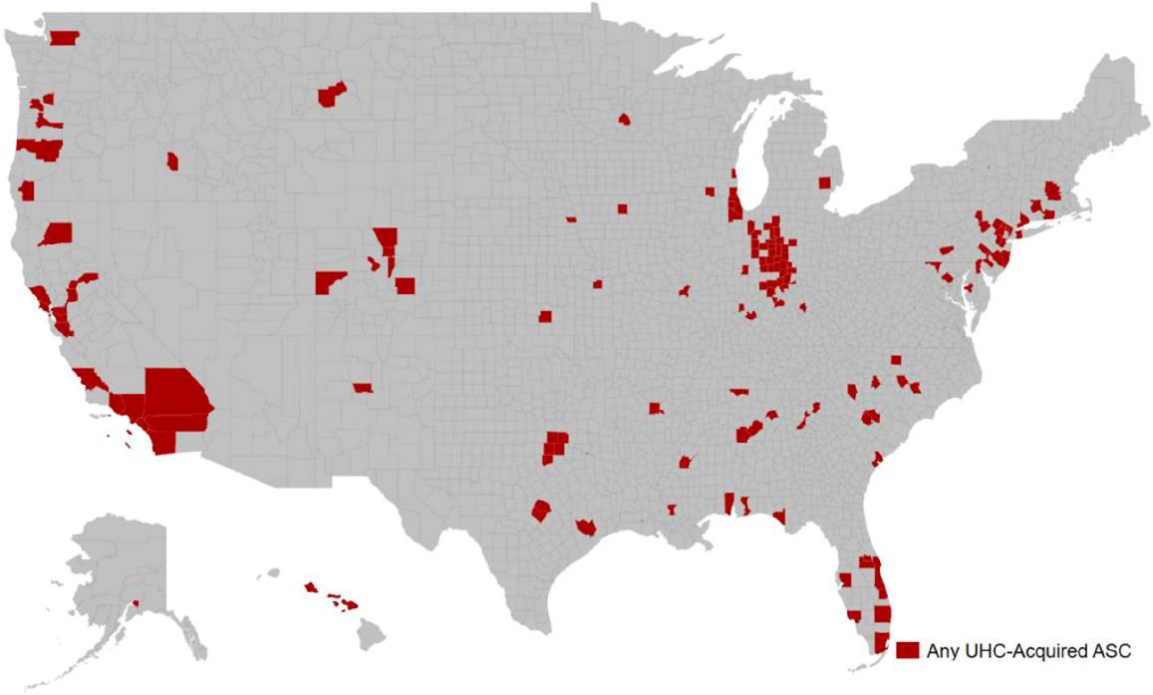
Geographic variation in counties with any ASC acquired by UnitedHealth. Source: Authors' analysis of data on ASC ownership obtained from an FOIA request and UnitedHealth SEC 10-K fillings on relevant business subsidiaries. The map shows the geographical distribution of UHC-owned ASCs in 2020. Abbreviations: ASC, ambulatory surgery center; FOIA, Freedom of Information Act; SEC, Securities and Exchange Commission; UHC, United HealthCare.

Figure 2 maps the resulting geographic spread of UHC ownership in 2020 at the county level. The M&A activity results in UHC owning ASCs in 147 counties across 35 states. However, the ASCs are not scattered uniformly across the country. The states with the largest number of counties with an acquired ASC are Indiana, California, and Florida. Figures S1 and S2 map the county-level number of ASCs and the share of total ASCs acquired by UHC, respectively, which show similar patterns.

Panel A in Table 1 presents the summary statistics of UHC insurance market share in counties with vs without UHC ASC presence. As the entry decision and pricing strategy might be different for commercial insurance plans and Medicare Advantage plans, we measured UHC insurance market share separately for the 2 segments. The average market shares are very similar in counties with and without UHC ASCs, both in the commercial market and the MA market.

**Table 1.** Characteristics of insurance markets and provider markets by UHC ASC presence.

	Counties without UHC ASC		Counties with UHC ASC	
	Mean	SD	Mean	SD
Panel A: UHC market share				
Commercial insurance market	0.141	0.091	0.150	0.090
MA insurance market	0.244	0.223	0.279	0.199
Panel B: provider market concentration				
ASC market	2386	1945	983	838
HOPD market	3227	2177	2264	1567

Abbreviations: ASC, ambulatory surgery center; FOIA, Freedom of Information Act; HHI, Herfindahl-Hirschman Index; HOPD, hospital outpatient department; MA, Medicare Advantage; UHC, United HealthCare.

Source: Authors' analysis of data on ASC acquisition obtained from an FOIA request and insurer market share data from Clarivate. This table presents the summary statistics of county-level UHC market share in insurance markets and county-level ASC market and HOPD market concentration by UHC ASC presence. Market concentration is measured by HHI. The sample of UHC ASCs is restricted to 1 year before the acquisition.

Panel B in Table 1 presents an analogous summary statistics of outpatient market concentration, stratified by UHC ASC presence. On average, counties served by UHC ASCs have lower levels of ASC market concentration and HOPD market concentration—signaling more competition among providers.

**Figure 3.**
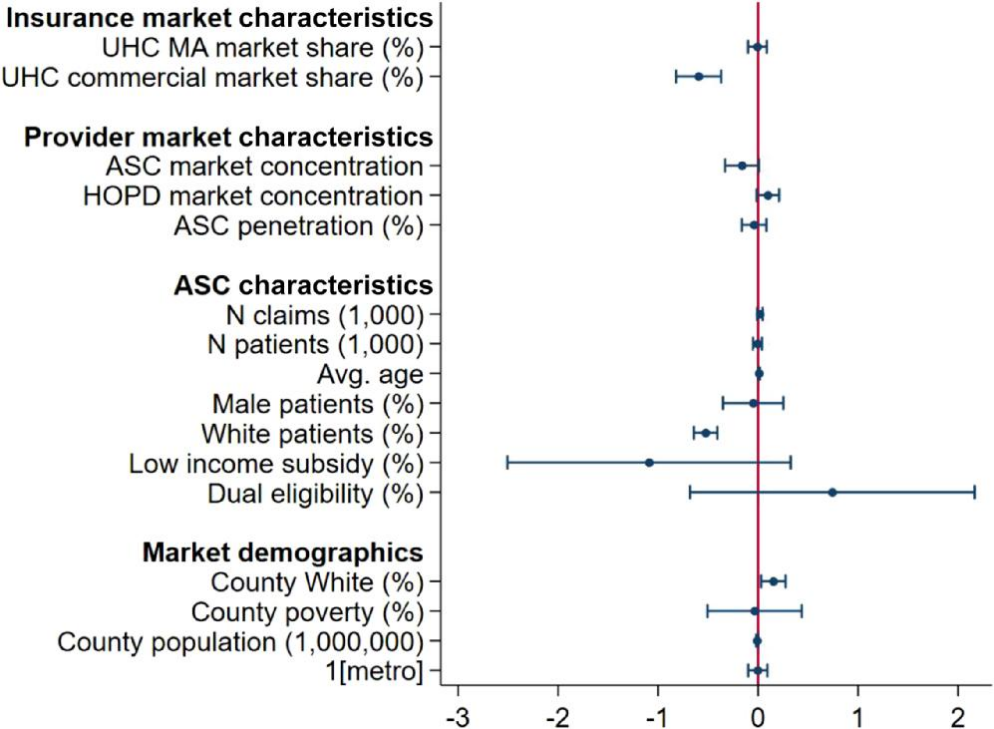
ASC and market characteristics associated with UHC ASC acquisition. Source: Authors' analysis of data on ASC acquisition obtained from an FOIA request and 100% sample of Medicare FFS claims data. This figure presents the estimated margins and standard errors of the multivariate analysis. The sample of UHC ASCs is restricted to 1 year before the acquisition. Abbreviations: ASC, ambulatory surgery center; Avg, average; FFS, fee-for-service; FOIA, Freedom of Information Act; HOPD, hospital outpatient department; MA, Medicare Advantage; UHC, United HealthCare.

Figure 3 offers results from a multivariate logistic regression at the ASC level to assess any localized but systematic pattern between newly UHC-owned ASCs and where they operate. After we introduced individual ASC characteristics and county demographics, we found that ASC market concentration tended to be negatively associated with acquisitions but statistically nonsignificant. UnitedHealthcare's commercial insurance market share was found to be statistically significantly associated with acquisitions along with other factors. For ASC characteristics, acquired ASCs provided similar number of procedures and served a similar number of Medicare beneficiaries. For patient mix, acquired ASCs served fewer White patients and a slightly older Medicare population than non–UHC-owned ASCs, although their respective patient mix was otherwise similar. Additionally, the county's White population was positively associated with where these newly UHC-owned ASCs tended to be located, although the overall population was slightly negatively associated.

The UHC–SCA transition is the largest one among all the ASC acquisitions. To test whether the SCA ownership affects the correlation, we repeated the multivariate analysis separately for ASCs that were previously owned by SCA and those that were not. As shown in Figures S3 and S4, we found similar correlations between ASC acquisition and insurance market share as well as provider market concentration.

## Discussion

In the US health care system, insurers and providers have historically operated independently for the most part. Insurer acquisition of providers is a new and emerging form of health care consolidation. This form of consolidation may have benefits, such as improved care coordination through aligned clinical and financial incentives, but may also increase market concentration and alter contract negotiation leverage within the multipayer landscape. To date, the patterns of insurer–provider consolidation are largely unstudied.

To begin to understand this new form of consolidation, this paper focused on insurer takeovers of ASCs, exploring trends in the acquisition of ASCs by the most dominant private insurer in the country, UHC. At a high level, acquisitions of ASCs tended to more frequently occur where UHC's commercial and Medicare Advantage market shares were stronger. However, when using a simple linear prediction model with adjustments for ASC and local market factors, the relationship between UHC’s insurance market position and its ASC targets became more complex. Ambulatory surgery centers acquired by UHC also tended to be associated with more competitive ASC markets, all else being equal. Simple heuristics, such as local levels of insurer or ASC competition, are therefore unlikely to sufficiently inform whether this form of consolidation is anticompetitive—at least according to the activity to date. However, the contemporary landscape does not necessarily predict how things will evolve over time, and future research will need to examine the post-acquisition behavior of ASCs in terms of services, payer mix, and pricing—especially in relation to non-UHC patient–payer combinations. Complex dynamics and interactions with insurer rules and specific markets are also possible, such as compliance with medical loss ratios and activity within the increasingly prominent Medicare Advantage market. Future research should examine these and a host of related important questions.

Insurer–provider consolidation appears to be the next wave of market consolidation in the United States. Previous iterations, such as hospital horizontal consolidation and hospital–physician vertical integration, often occurred before regulators could adequately respond to preserve market competition. While most individual health care transactions are too small to trigger regulator review, the cumulative impact can lead to harmful market concentration. The current and emerging trend of provider–insurer integration poses a new challenge for regulators and policymakers. Meeting the challenge requires both transparency on acquisitions and robust research on the consequences of this new form of integration.

## Supplementary Material

qxae081_Supplementary_Data
