# Supplementary material for: Market landscape and insurer–provider integration: the case of ambulatory surgery centers
Source: Health Aff Sch. 2024 Jun 11;2(6):qxae081. doi: 10.1093/haschl/qxae081 (PMC11195573; doi:10.1093/haschl/qxae081)

Supplemental Appendix

Figure S1: Geographic Variation in County-Level Number of ASCs Acquired by UnitedHealth

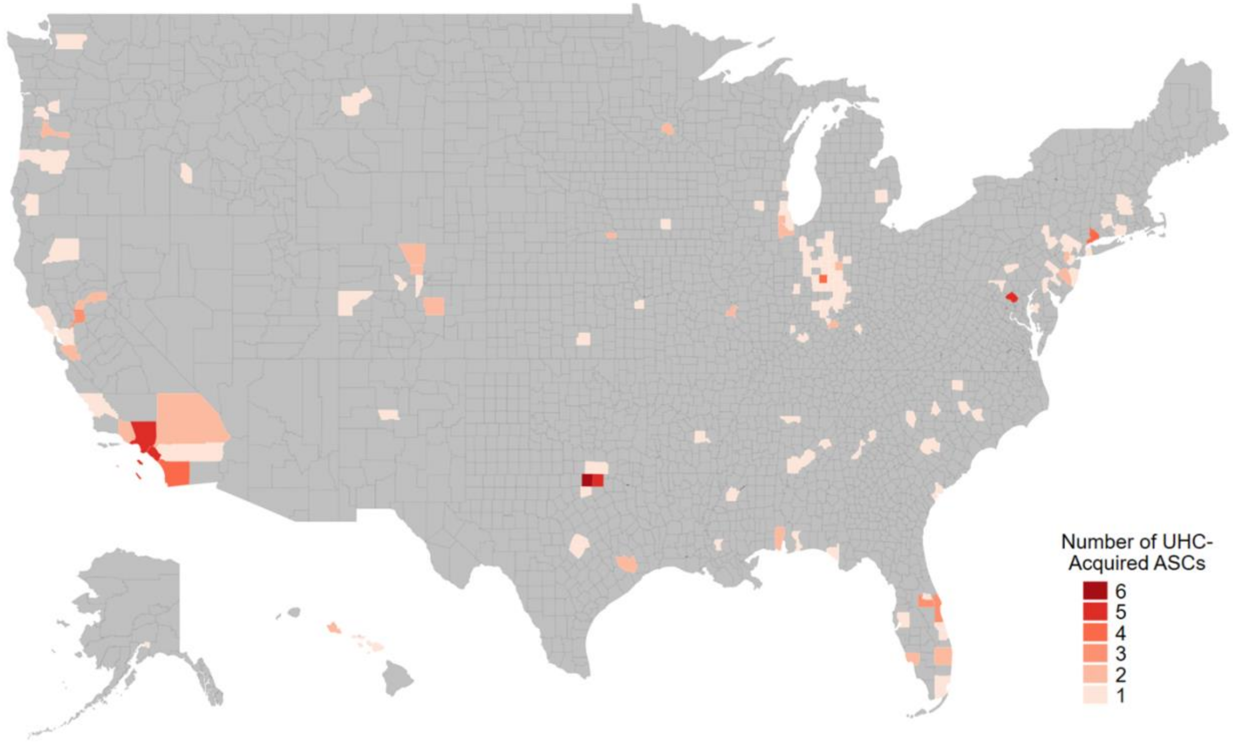

Supplement: qxae081_Supplementary_Data [file qxae081_supplementary_data.zip › FigureS1.pdf]
