# Supplementary figures and images for: Market landscape and insurer–provider integration: the case of ambulatory surgery centers
Source: Health Aff Sch. 2024 Jun 11;2(6):qxae081. doi: 10.1093/haschl/qxae081 (PMC11195573; doi:10.1093/haschl/qxae081)

**Figure S2: Geographic Variation in County-Level Share of ASCs Acquired by UnitedHealth (2020)**

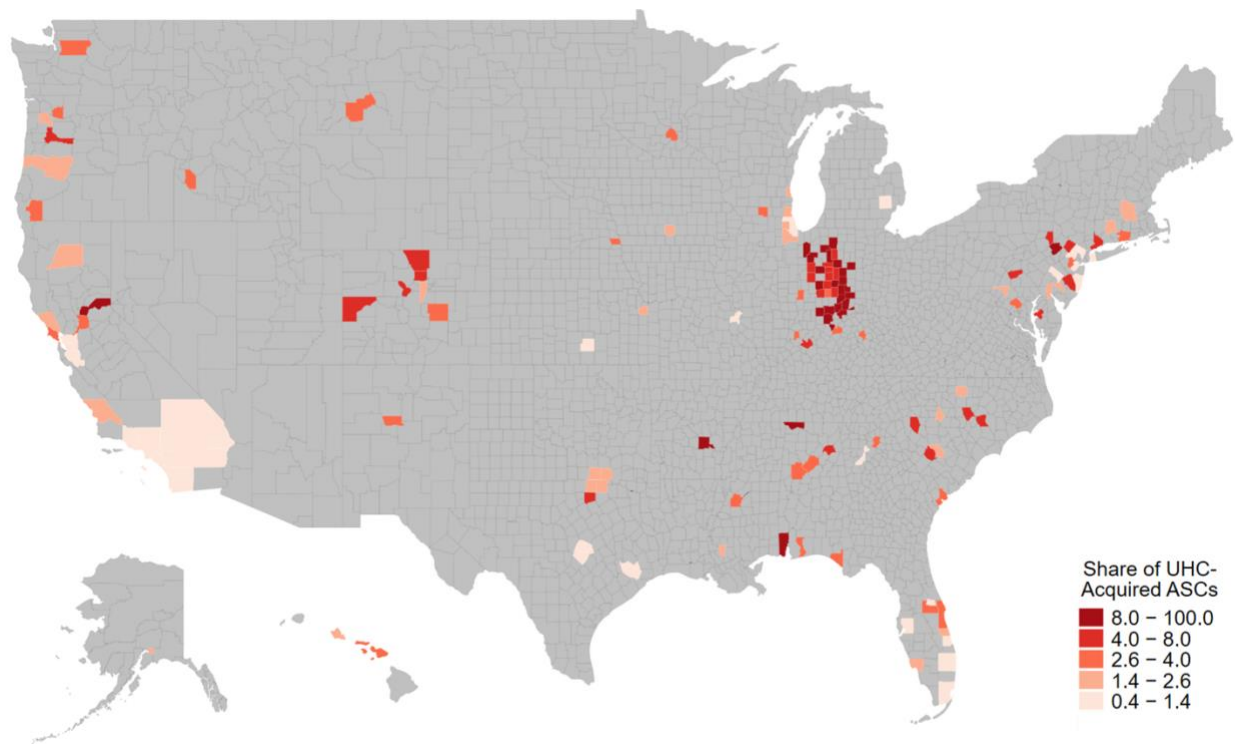

Supplement: qxae081_Supplementary_Data [file qxae081_supplementary_data.zip › FigureS2.pdf]

**Figure S3: ASC and Market Characteristics Associated with UHC-SCA Acquisition**

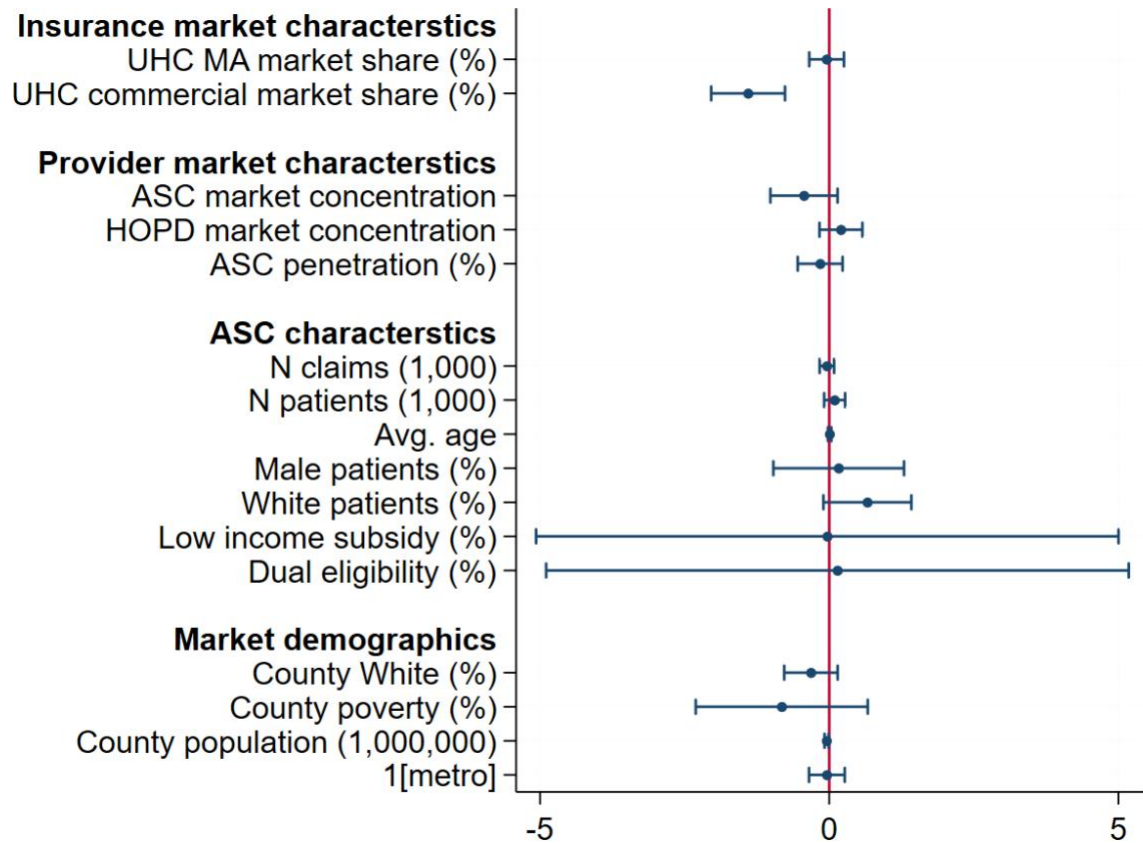

Supplement: qxae081_Supplementary_Data [file qxae081_supplementary_data.zip › FigureS3.pdf]

**Figure S4: ASC and Market Characteristics Associated with UHC non-SCA ASC Acquisition**

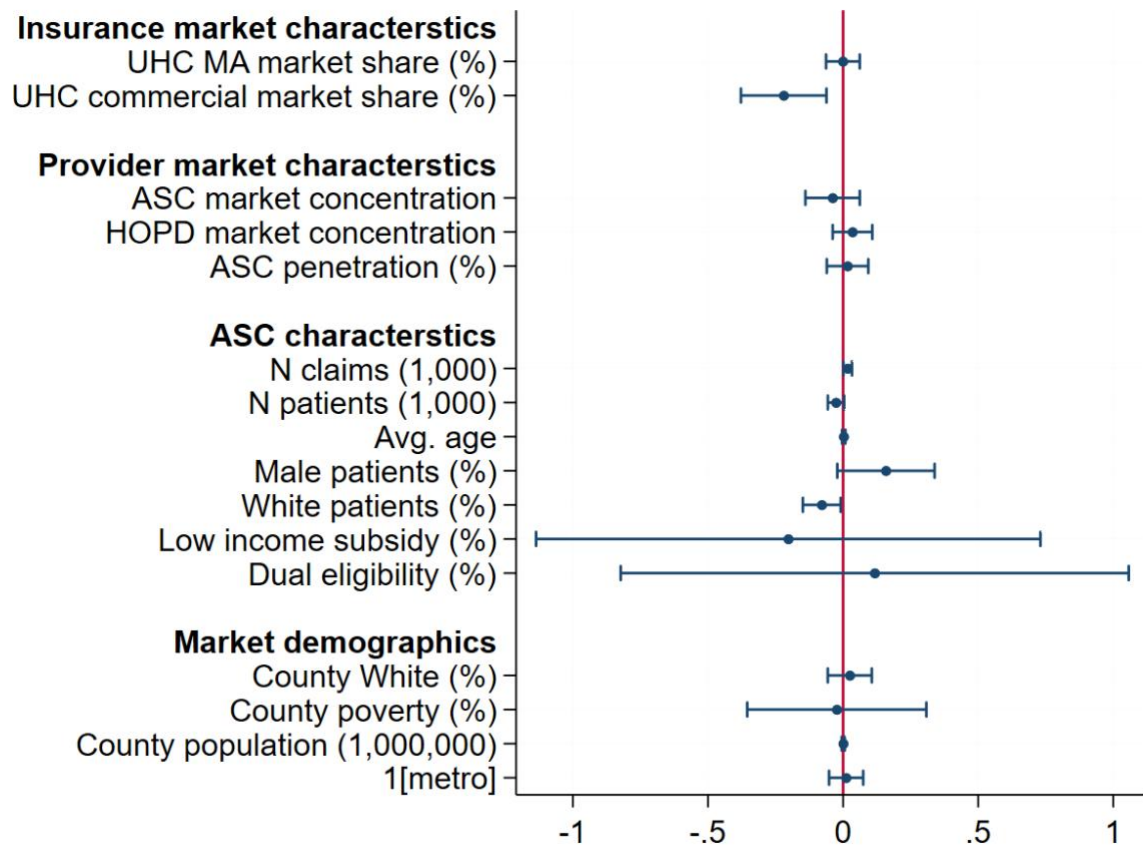

Supplement: qxae081_Supplementary_Data [file qxae081_supplementary_data.zip › FigureS4.pdf]
